# Massive Water Production from Cryogenic Icy Lunar Regolith by a Microwave Heating Method

**DOI:** 10.34133/research.0800

**Published:** 2025-07-30

**Authors:** Qinggong Wang, Yong Pang, Juping Gu, Zhongxian Zhao, Qichen Sun, Mengfei Yang

**Affiliations:** ^1^ School of Energy and Environmental Engineering, University of Science and Technology Beijing, Beijing 100083, P. R. China.; ^2^ Qian Xuesen Laboratory of Space Technology, China Academy of Space Technology, Beijing 100094, P. R. China.; ^3^ Beijing Spacecrafts, China Academy of Space Technology, Beijing 100094, P. R. China.; ^4^ China Academy of Space Technology, Beijing 100094, P. R. China.

## Abstract

Water extraction from lunar regolith is one of the critical techniques for in situ resource utilization on the Moon. Traditional conductive heating methods show low efficiencies for massive water production from lunar regolith due to the low heat conductivity of lunar regolith and the inherent harsh lunar environment. To enhance the effectiveness of massive water production and reduce energy cost, microwave heating method is used in this study to extract water from icy lunar regolith at an initial cryogenic temperature of −80 °C. Hydrated lunar regolith simulant (LRS) is prepared with the water content from 1.96% to 13.79%. The particles are compacted into large cylinder sample with a size of 70 mm (diameter) × 70 mm (height). An integrated microwave heating system is built by which the icy lunar regolith is heated by microwaves at 2.45 GHz. Water vapor is transmitted in a closed flow path, and liquid water is collected in a cold trap after condensation. Both a high-power heating pattern (800 W) and a low-power heating pattern (400 W) are studied. The results show that microwave heats the sample uniformly, and water content escapes from center to outside. At the high power of 800 W, the samples are dried almost completely at an energy cost of 1.9 to 10.0 W·h/g with the decrease of water content. The rate of water collection is up to 1.57 g/min. Reducing heating power to 400 W prolongs the initial heat input period. Once the liquid water begins to collect, the water collection rate is comparable to that at the high-power heating pattern. The results prove the effectiveness of microwave heating for massive water production from icy lunar regolith, and the technique can be used for future engineering applications.

## Introduction

Water extraction from lunar regolith has become a hot topic in recent years, particularly after the return of China’s Chang’E-5 lunar regolith from the near side of the Moon [1,2], and the Chang’E-6 lunar regolith from the far side [3]. Water is the most important resource for human’s presence on the Moon and further deep space exploration [4–6]. To date, there have been many evidences of water existence on the Moon, which can be divided into 2 groups: remote sensing from orbiting spacecraft, and laboratory investigation of lunar samples returned from the Moon [7,8]. The former studies were started from 1990s, which mainly confirmed the presence of water ice in permanently shadowed regions (PSRs) near the lunar poles. In 1994, the instrument onboard NASA lunar orbiter Clementine mapped the Moon for the first time and discovered possible existence of ice within some of the Moon craters [9,10]. In 1998, the neutron spectrometer instrument onboard Lunar Prospector (LP) detected hydrogen, which was assumed to be in the form of water ice. The acquired data indicated that a large quantity of water ice was mixed into the regolith at the poles [11]. In 2008, the reflectance spectroscopy on Chandrayaan-1 detected surface correlated OH/H_2_O species in lunar soil at low latitudes [12,13]. Until 2018, the results of Moon Mineralogy Mapper (M^3^) instrument on the Chandrayaan-1 showed direct evidence of water ice trapped in PSRs of the Moon [14]. In 2009, Lunar Reconnaissance Orbiter (LRO) and Lunar Crater Observation and Sensing Satellite (LCROSS) missions performed a controlled impact experiment, and the results indicated that the large quantities of volatiles included about 5.6 ± 2.9 wt % water [15,16]. Recently, NASA/DLR Stratospheric Observatory for Infrared Astronomy (SOFIA) revealed a 6-μm emission feature at high lunar latitudes. It indicated the presence of molecular water with an abundance of about 100 to 400 μg/g H_2_O [17].

On the other hand, laboratory investigation of lunar samples provide more direct evidences of water existence on the Moon [18]. Previously, the Moon was thought to be an anhydrous body, because the laboratory-based analysis of Apollo samples indicated a near absence of any water-bearing mineral phases in Moon rocks. The occurrence of Fe metal and the lack of Fe^3+^ in lunar minerals provided strong evidence for the absence of water in the lunar interior [19]. It is only in recent decades that technological advancements in modern analytical instrumentation have permitted unambiguous detection of water and other volatiles in lunar samples [18]. In 2008, Saal et al. [20] provided the first direct absolute measurement of water in lunar samples from analysis of volcanic glasses. They used the secondary ion mass spectrometry (SIMS) instrument and found that the high-Ti orange glasses contained approximately 5 to 13 parts per million (ppm) H_2_O, while the very-low Ti green glasses were characterized by H_2_O contents of 0.4 to 30 ppm. Later, McCubbin et al. [21] and other research groups [22,23] used ion-microprobe techniques to directly detect and quantify hydroxyl in lunar apatites. A wide range of water contents have been reported, from few ppm levels to several thousands of ppm. In 2012, Liu et al. [24] made a direct measurement of hydroxyl in the Apollo samples by Fourier transform infrared spectroscopy (FTIS) and SIMS analyses. The retention of solar wind-produced OH in these samples supported solar wind as a viable source for water ice in polar cold traps. In 2013, Hui et al. [25] analyzed plagioclase grains in lunar anorthosites of the Apollo samples. They estimated that the initial water content of the lunar magma ocean is approximately 320 ppm, and water accumulating in the final residuum of the lunar magma ocean could have reached 1.4 wt %. In recent years, many studies have been performed on the Chang’E-5 sample. Lin et al. [26] analyzed the reflectance spectra acquired by the Chang’E-5 lander, and estimated up to 120 ppm of water (OH + H_2_O) in the lunar regolith and about 180 ppm of water in the rock. Zhou et al. [27] performed spectral and microstructural analyses for the Chang’E-5 sample. They estimated a minimum of 170 ppm water content in lunar soils in the Chang’E-5 region and found that solar wind-derived water was affected by exposure time, crystal structure, and mineral composition. Liu et al. [28] provided further evidences of water on the lunar surface from both Chang’E-5 in situ spectra and the returned samples. They showed that hydroxyl contents were in the range of 0 to 179 ± 13 ppm at the young mare region. Jin et al. [29] presented the discovery of a hydrated mineral, (NH_4_)MgCl_3_·6H_2_O, in lunar soil samples returned by Chang’E-5 that contains approximately 41 wt % H_2_O. Lin et al. [30] presented a result of a higher water content in the fine fractions than in bulk soil and coarse fractions. Water was predominantly concentrated in the outermost rims of the regolith grains, indicating that solar wind is a primary source of lunar surface water. Zhou et al. [31] reported large amounts of OH and molecular H_2_O related to solar wind and other multiple sources preserved in impact glasses from Chang’E- 5 lunar soil based on reflectance infrared spectroscopy and NanoSIMS analyses. The results revealed that impact glasses were the main carrier of molecular H_2_O in lunar soils. Tian et al. [32] measured H abundances and D/H ratios on soil grains from 3 deepest sections of the Chang’E-5 drill core sampled at depths of 0.45 to 0.8 m. High water contents of 0.13 to 1.3 wt % were present on approximately half of the grain surfaces (topmost ∼ 100 nm), comparable to the values of Chang’E-5 scooped soils. These results provided evidence that the lunar regolith below the surface contained considerable water from solar wind implantation.

After water detection, the next challenge is to extract it from lunar regolith. However, the harsh environment of lunar surface [33,34] and unclear hydration of water ice with regolith [35–38] bring difficulties to the in situ water extraction. Particularly, massive water production is challenging. Several thermal methods have been proposed previously, by which the icy lunar regolith is heated conductively and water ice is driven to sublimate [39–46]. In this context, Zacny et al. [39] proposed a “Planetary volatiles extractor (PVEx)” by combining regolith mining and water extraction. The water efficiency of the PVEx was over 50% from the regolith at an initial water content of 5%. Reiss et al. [40] used a small ceramic heater to thermally extract volatiles from the lunar regolith simulant (LRS). Water was in a fraction of around 0.1% to 0.2% and heated from −50 to 300 °C. Purrington et al. [45] used a laboratory lamp to heat the surface of icy regolith. This method only desiccated the surface sample, resulting in a sublimation rate of water ice less than 1.0 g/h. Liu et al. [46] built a pilot-scale apparatus based on a drilling–heating method. The icy regolith was first drilled and collected into the drill chamber, and then heated by the drill rod. The heating efficiency was higher than previous methods, and the water collection rate was up to 0.75 g/min at an energy cost of 8.6 to 37.9 W·h/g. It was widely found in these traditional heating methods that the water extraction processes are energy intensive and sometimes inefficient due to the low conductivity of lunar regolith [47–49]. It is hard to extract the crystallized water because the heating temperature should be over 200 °C.

To improve the water extraction efficiency, microwave heating is potentially a better method. This is because microwave energy can excite the polar molecules in materials and transmits to water molecules directly. The samples can be heated uniformly despite of their volumes. Microwave energy penetrates into the soil and extract water removal from deep below the lunar surface. Until now, the results on water extraction from lunar regolith by microwave heating are quite limited [50–52]. This method is very immature and far from applicable to massive water production from icy lunar regolith for engineering applications. In this study, we investigate the microwave heating method for water extraction from icy lunar regolith and focus on the massive water production from a large amount of hydrated lunar regolith simulant (LRS). An integrated microwave heating system is built, by which the compacted icy LRS sample is heated intensively and the escaped water vapor is collected in a cold trap. The process of massive water production is recorded in the experiment. Water yield ability and energy cost of the present microwave heating unit are demonstrated. The findings can be used for future engineering application of microwave heating to produce water massively from icy lunar regolith.

## Results and Discussion

### High-power heating pattern

**Fig. 1.**
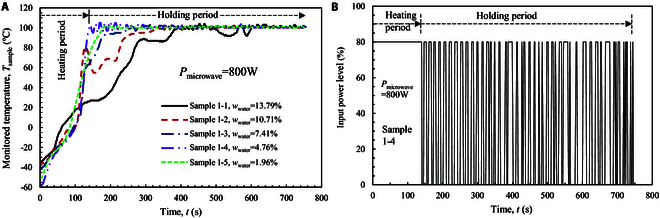
Microwave heating process of the samples at a heating power of 800 W. (A) Transient temperature rise of the sample. (B) Microwave heating pattern for sample 1-4.

Heating power is a key parameter that determines the heating intensity of the microwave. A high power of 800 W is tested first in the experiments. Each sample is heated at a heating temperature of 100 °C and a total heating time of 750 s. The experimental results are given in Table 1. Figure 1A shows the transient temperature rise of the sample (*T*_sample_) as monitored by the Pt100 sensor during microwave heating. The total heating time length is divided by 2 different periods: heating period (*t*_heating_) and holding period (*t*_holding_). In the heating period, the microwave power is input continually (see Fig. 1B). The temperature of the icy LRS sample increases quickly from the cryogenic condition to the set temperature of 100 °C. As shown in Fig. 1A, the initial temperature of the sample is between −30 and −60 °C for the samples. This measured temperature departs from the initial temperature of −80 °C. This is because when the sample is taken out from the refrigerator and in contact with the atmosphere, the temperature at the inner surface of the drilled hole increases. At the same time, there exists a thermal resistance between the inner surface of the sample and the temperature sensor. Some time is required to reach thermal balance to measure the temperature timely. When the temperature is over 0 °C, time delay of the monitored temperature almost diminishes.

**Table 1.** Experimental cases and the measured results

Sample	Initial water content, *w*_water_ (-)	Microwave power, *P*_microwave_ (W)	Heating temperature, *T*_heating_ (°C)	Heating time, *t*_heating_ (s)	Holding time, *t*_holding_ (s)	Initial weight of sample, *G*_0_ (g)	Final weight of sample, *G*_f_ (g)	Initial weight of water, *m*_0,water_ (g)	Water extracted, *m*_1_*,*_water_ (g)	Extraction ratio, *γ*_1_,_water_ (-)	Power input in heating period, *W*_heating_ (kJ)	Power input in holding period, *W*_holding_ (kJ)	Total power input, *W*_total_ (kJ)	Water absorption power, *W*_water_ (kJ)	LRS absorption power, *W*_LRS_ (kJ)	Power absorption ratio by water, *Γ*_water_ (-)	Power absorption ratio by LRS, *Γ*_LRS_ (-)	Energy cost, *η* (W·h/g)	Water collected, *m*_2_*,*_water_ (g)	Collection ratio, *γ*_2_,_water_ (-)	Collection rate, *ψ*_water_ (g/min)
1-1	13.79%	800	100	385	365	502.2	435.8	69.3	66.4	95.7%	308.0	153.6	461.6	177.7	84.6	38.5%	18.3%	1.9	23.0 ^a^	34.6% ^a^	1.57
1-2	10.71%	800	100	332	419	509.6	458.1	54.6	51.5	94.2%	265.6	174.4	440.0	137.8	88.9	31.3%	20.2%	2.4	17.5	34.0%	1.14
1-3	7.41%	800	100	262	499	508.3	474.3	37.7	34.0	90.3%	209.6	148.8	358.4	91.0	92.1	25.4%	25.7%	2.9	11.5	33.8%	0.84
1-4	4.76%	800	100	140	610	507.3	484.7	24.2	22.6	93.6%	112.0	204.8	316.8	60.5	94.1	19.1%	29.7%	3.9	5.5	24.3%	0.35
1-5	1.96%	800	100	179	571	508.3	498.9	10.0	9.4	94.3%	143.2	195.2	338.4	25.2	96.9	7.4%	28.6%	10.0	3.8	40.4%	0.33
2-1	10.71%	400	100	724	26	508	466.8	54.4	41.2	75.7%	289.6	8.0	297.6	110.2	90.6	37.0%	30.5%	2.0	12.0	29.1%	0.71
2-2	4.76%	400	100	568	182	500	479.7	23.8	20.3	85.3%	227.2	28.0	255.2	54.3	93.1	21.3%	36.5%	3.5	6.5	32.0%	0.61
2-3	1.96%	400	100	438	312	495.4	486.7	9.7	8.7	89.6%	175.2	55.2	230.4	23.3	94.5	10.1%	41.0%	7.4	4.2	48.3%	0.39

^a^ In this case, water vapor leaks to the environment because of inadequate condensation efficiency in the condenser.

Figure 1A shows that the time length *t*_heating_ and the rate of temperature rise are both dependent on the initial water content (*w*_water_). The heating period (*t*_heating_) reduces from 385 s to 179 s when *w*_water_ decreases from 13.79% to 1.96% (see Table 1). When *w*_water_ is over 10%, the temperature rise shows a retard phenomenon at some temperature, e.g., at about 20 °C for sample 1-2 and about 65 °C for sample 1-1. During this retarding period, heat is accumulated to melt water ice to liquid. The temperature stops to rise until adequate heat is absorbed for liquid evaporation. After this period, *T*_sample_ increases quickly again. Once *T*_sample_ reaches the setting temperature, it enters into the holding period (*t*_holding_). In the holding period, the temperature is fixed at 100 °C, and microwave is input intermittently (see Fig. 1B). Within the fixed total heating time of 750 s, the time length of *t*_holding_ increases with the decrease of *w*_water_ (see Table 1).

The amount of power input in each period is calculated based on the data of Fig. 1B. The power input in heating period (*W*_heating_) is obtained by multiplying the microwave power (*P*_microwave_) and the heating time length (*t*_heating_). The power input in holding period (*W*_heating_) is the product of *P*_microwave_ and the net heating time in the holding period (see Fig. 1B). It shows in Table 1 that the total power input decreases from 461.6 kJ to 338.4 kJ when *w*_water_ decreases from 13.79% to 1.96%. Due to the decrease of water content, the amount of heat absorption by LSR sample is reduced. The power input in the heating period (*W*_heating_) is the main contribution for water evaporation when *w*_water_ ≥7.41%, while that in holding period (*W*_holding_) becomes dominant when *w*_water_ ≤4.76%. After microwave heating, the sample is dried almost completely, because over 90% of water content has been extracted from the sample at the high power of 800 W.

The amount of extracted water (*m*_1,water_) is calculated from the weights of the sample before and after microwave heating, as

$$m_{1,\text{water}}=G_{0}-G_{f}$$ (1)

where *G*_0_ and *G*_f_ is the initial and final weights of the sample, respectively.

The initial water weight (*m*_0,water_) is estimated from the water content (*w*_water_) as

$$m_{0,\text{water}}=G_{0}\cdot w_{\text{water}}$$ (2)

The water extraction ratio (*γ*_1,water_) is

$$\gamma_{1,\text{water}}=m_{1,\text{water}}/m_{0,\text{water}}$$ (3)

**Fig. 2.**
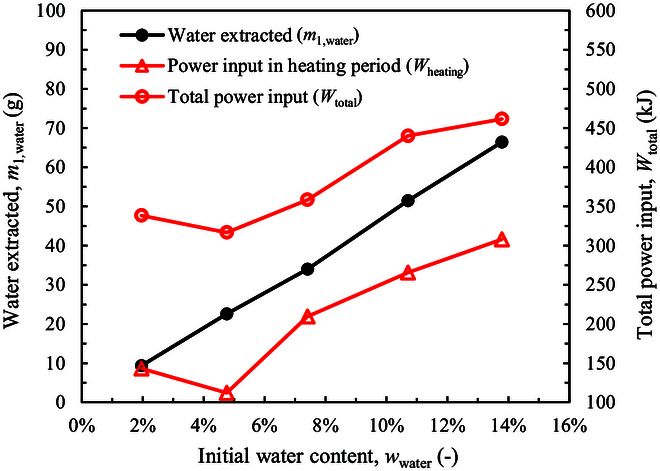
Relationship between water extracted and power inputs.

As shown in Table 1, over 90% to 95% of water is extracted at the high power and with an adequate heating time of 750 s. Figure 2 shows that with a decrease of water content (*w*_water_), the amount of water extracted (*m*_1,water_) decreases linearly. *m*_1,water_ is strongly related with the power inputs (*W*_heating_ and *W*_total_). This is because when the water content is small, microwave heating becomes less efficient due to the low absorption property of the regolith [53–55]. Both *W*_heating_ and *W*_total_ decrease. Although *t*_holding_ is prolonged, *W*_holding_ is not enhanced substantially (see Table 1).

To evaluate the heat absorption efficiency, the amounts of energy absorbed by water and LRS are calculated. The power absorbed by water (*W*_water_) is obtained by

$$W_{\text{water}}=\left(h_{\text{vapor}}-h_{\mathrm{ice}}\right)m_{1,\text{water}}$$ (4)

where *h*_vapor_ is the enthalpy of vapor at the operating pressure, *h*_ice_ is the enthalpy of ice at the initial temperature, and *m*_1,water_ is the mass of water extracted. Here, we use the enthalpy of saturated vapor at 1.0 atm for *h*_vapor_, as 2,675.53 kJ/kg, and *h*_ice_ is close to zero due to the deep low temperature of −80 °C.

The power absorbed by the LRS (*W*_LRS_) is

$$W_{\mathrm{LRS}}=C_{p,\mathrm{LRS}}G_{f}\left(T_{\text{heating}}-T_{0}\right)$$ (5)

where *C*_p,LRS_ is the specific heat of the LRS, as given in the section “Lunar regolith simulant”. *G*_f_ is final weight mass of the LRS, *T*_heating_ is the heating temperature, and *T*_0_ is the initial temperature of the LRS.

**Fig. 3.**
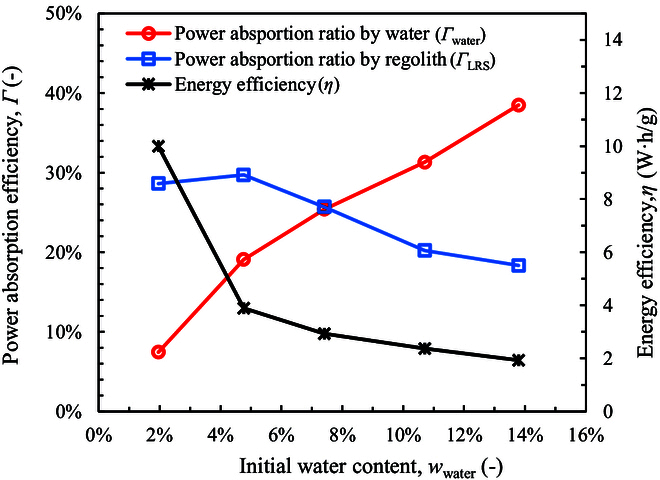
Power absorption ratios by water and regolith.

The power absorbed by water (*W*_water_) and by regolith (*W*_LRS_) are given in Table 1. Compassion between the power absorption ratio and the water content is shown in Fig. 3. When the initial water content (*w*_water_) decreases from 13.79% to 1.96%, the ratio of power absorbed by water decreases almost linearly from 38.5% to 7.4%. The power absorbed by LRS increases slightly from 18.3% to about 29%. The power absorbed by LRS is assumed to be conducted from molecular water due to the low microwave absorption ability of the LRS particles. The rest power ratio is about 43.2% to 64.0%, which is absorbed by the SiC pieces at the bottom of the sample cylinder (see “Experimental system”) and is lost to the environment. As a result, the energy cost of microwave heating (*η*) increases with the decrease of *w*_water_. The *η* value increases only from 1.9 to 3.9 W·h/g when *w*_water_ decreases from 13.79% to 4.76%. This cost is much lower than the traditional heating method as reported in the previous work [46] where the energy cost is about 8.6 W·h/g for the water content of 6% under the similar cryogenic condition. However, when *w*_water_ is as low as 1.96%, the energy cost increases significantly to 10.0 W·h/g. Even so, it is much lower than that of the traditional heating method, which is about 37.9 W·h/g for a water content of 2% [46].

**Fig. 4.**
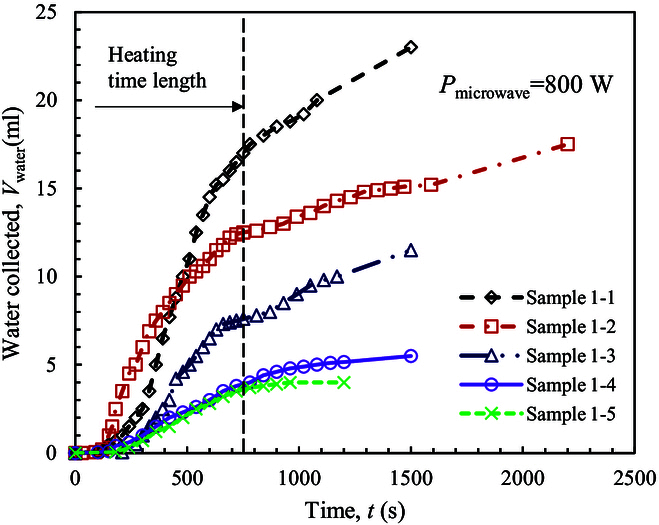
Transient water collection at the heating power of 800 W.

The transient water collection process, as recorded by the camera, is shown in Fig. 4. Once microwave heating starts, water ice in the LRS sample melts and evaporates quickly. Due to the long transport path of vapor in the collection system (about 1 m; see “Experimental system”), the first drop of liquid water is collected at about 100 s when *w*_water_ > 4.76% (i.e., samples 1-1, 1-2, and 1-3), and it postpones to about 200 s when *w*_water_ ≤ 4.76% (i.e., samples 1-4 and 1-5). After this time point, water vapor is condensed quickly. Microwave heating stops at the end of the heating time of 750 s. Afterward, the air pump still works to collect vapor in the furnace and the rest water liquid in the tube of flow path. The final weight of water collected (*m*_2,water_) is obtained at the end of experiment. The water collection ratio (*γ*_2,water_) is calculated by

$$\gamma_{2,\text{water}}=m_{2,\text{water}}/m_{1,\text{water}}$$ (6)

where *m*_1,water_ is the weight of water extracted.

As seen from Table 1, the water collected (*m*_2,water_) decreases from 23.0 g to 3.8 g when *w*_water_ decreases from 13.79% to 1.96%. The water collection ratio (*γ*_2,water_) is about 30% to 40%. The rest water vapor is leaked to environment due to 2 reasons. In the present system, the evaporated vapor is transported by air pump. The positive pressure forces water vapor to transport, and vapor leaks from the slit of the furnace during transportation. Besides, water vapor is generated massively when the water content is high (e.g., *w*_water_ = 13.79%). In this case, the condensation efficiency of the ball condenser is not adequate to condense all vapor, and a fraction of water vapor leaks to environment from the collector. Even so, massive water production is well achieved by the present system. The averaged water collection rate (*ψ*_water_) is calculated in the time length between the time point of first drop of water collected and the end of microwave heating period. The value is about 0.84 to 1.57 g/min when *w*_water_ is above 7.41%, and it decreases to about 0.35 g/min when *w*_water_ is below 4.76%.

**Fig. 5.**
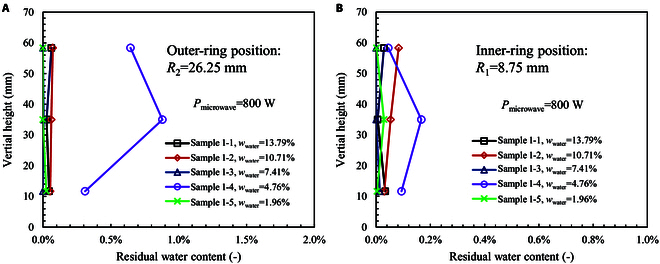
Vertical distribution of residual water content in the sample after microwave heating. (A) Outer-ring position (*R*_1_). (B) Inner-ring position (*R*_2_). The heating power is 800 W.

After microwave heating, the rest moisture in the sample is measured. The sample is decomposed into 6 zones following the strategy given in the “Experimental system”. As shown in Fig. 5, the residual water contents in all 5 samples are very low. For samples 1-1, 1-2, 1-3, and 1-5, the residual water content is below 0.1% in both the outer-ring position (*R*_2_) and inner-ring position (*R*_1_), and it is distributed uniformly along the vertical direction. Thus, the samples are completely dry at the high power of 800 W. For sample 1-4, the outer-ring position (*R*_2_) has a residual water content of about 0.5% to 1.0%, while the inner-ring position (*R*_1_) has a low water content below 0.2%. This indicates that the sample is heated from center by microwave. Water vapor escapes from center and migrates to outside. This heating pattern is different from the traditional conductive heating method [39–43], and it shows a much higher heating efficiency. In the middle level (*L*_2_), the residual water content is higher than both the top level (*L*_1_) and bottom level (*L*_3_). This is because water vapor in the middle level has a higher resistance to escape from the sample because of the longer vapor transport path.

### Low-power heating pattern

**Fig. 6.**
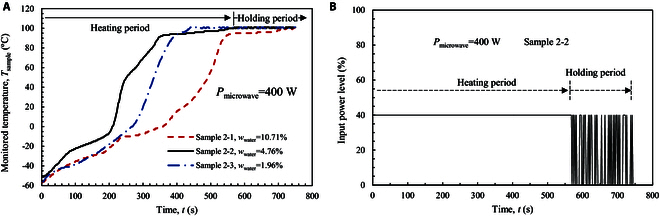
Microwave heating process of the samples at a heating power of 400 W. (A) Transient temperature rise of the sample. (B) Microwave heating pattern for sample 2-2.

Energy saving is an important problem for massive water production, particularly in the lunar environment. To obtain the water extraction efficiency at a low-power level, the heating power of microwave is reduced by half to 400 W in this section. The heating temperature is still fixed at 100 °C, and the total heating time is at 750 s. Figure 6A shows the transient temperature rise of the 3 samples at the low power (*P*_microwave_) of 400W. The heating period (*t*_heating_) is prolonged substantially, and the rate of temperature rise becomes slow. The *t*_heating_ value is 724 s for sample 2-1 with an initial water content (*w*_water_) of 10.71%, and it is 438 s for sample 2-3 with a low *w*_water_ of 1.96%. With the low heating power, the retard phenomenon of temperature rise becomes obvious, e.g., at about −10 °C for sample 2-1 and at about −20 °C for sample 2-2. A longer time is required for the sample to accumulate heat for icy melting and water evaporation, particularly when water content is high.

The heating pattern is changed correspondingly at the low heating power, as shown in Fig. 6B. The sample is heated continuously in the prolonged heating period, and it switches to intermittent mode in the holding period (*t*_holding_). The total power input (*W*_total_) is reduced at the low heating power to below 300 kJ for all 3 samples, and it decreases with *w*_water_ (see Table 1). The power input in the heating period (*W*_heating_) occupies a large fraction over 75% to 97%. As a result, the water extraction ratio (*γ*_1,water_) is about 75% to 90%, which shows an increase with *w*_water_. These values of *γ*_1,water_ are slightly lower than those obtained at the high power of 800 W. It is interesting to see that the power absorption ratios both by water (*Γ*_water_) and by regolith (*Γ*_LRS_) are higher than those at 800 W for the same sample. This indicates that heat is more effectively absorbed by the sample at the low heating power due to less energy lost to the environment. Under such conditions, the energy costs (*η*) reduce to 2.0, 3.5, and 7.4 W·h/g for samples 2-1, 2-2, and 2-3, respectively.

**Fig. 7.**
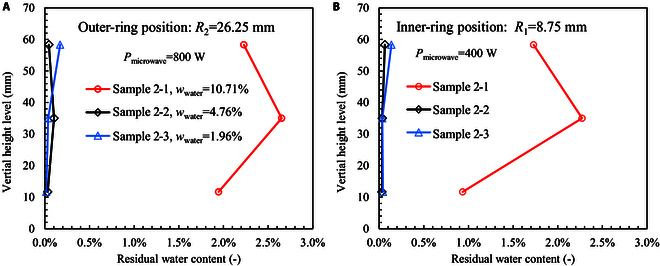
Vertical distribution of residual water content in the sample cylinder after microwave heating. (A) Outer-ring position (*R*_2_). (B) Inner-ring position (*R*_1_). The heating power is 400 W.

Figure 7 shows the residual water contents in the samples after microwave heating. For sample 2-2 with *w*_water_ = 4.76% and sample 2-3 with *w*_water_ = 1.96%, the residual water contents are below 0.2%. Thus, these 2 samples are dried completely even at a low power of 400 W. Differently, sample 2-1 with *w*_water_ = 10.71% shows obvious residual water contents, which are about 2.0% to 2.7% in the outer-ring position (*R*_2_) and about 1.0% to 2.5% in the inner-ring position (*R*_1_). Still, the middle level (*L*_2_) shows a higher water content than the other 2 levels because water vapor is difficult to escape from this zone. For this sample with a high initial water content of 10.71%, the total input power is not adequate to extract all the moisture. The water extraction ratio is about 75.7%, and the energy cost is as low as 2.0 W·h/g when the sample is not dried completely (see Table 1). A prolonged holding period is helpful to extract all water content, but the energy cost will be enhanced correspondingly.

**Fig. 8.**
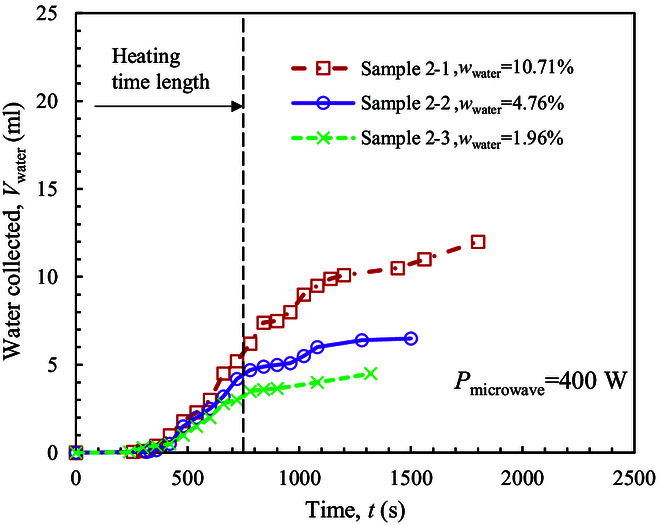
Transient water collection at the heating power of 400 W.

Figure 8 shows the transient water collection process at the heating power of 400 W. A long time of about 300 s is required before the first drop of liquid water is collected. Afterward, the volume of water collected increases almost linearly until the end of the heating time length. During this period, the average water collection rate (*ψ*_water_) is 0.71, 0.61, and 0.39 g/min for the 3 samples, showing a decrease with the decrease of *w*_water_. The final water collected ratio (*γ*_2,water_) is about 29% to 51.7%. It increases with the decrease of *w*_water_. Both *ψ*_water_ and *γ*_2,water_ values are comparable to those obtained at the high power of 800 W. Thus, the low heating power of 400 W is adequate to extract water from icy lunar regolith. Under the low-power heating pattern, the heat absorption efficiency by icy LRS is even higher. Although the first drop of liquid water is collected much later, the overall water production capability is not affected substantially in the following period. Thus, if the initial water content is high, the total heating time can be prolonged to extract adequate water resources.

## Conclusion

In this study, massive water production from icy lunar regolith is achieved by microwave heating. Both a high-power heating pattern and a low-power heating pattern are evaluated, and the following conclusions are obtained.

The LRS sample is heated uniformly by microwave. The temperature of the sample increases from −80 to 100 °C in the experiment. At the high power of 800 W, over 90% of water content is extracted, and the samples are dried almost completely with little water residue in the sample. When the initial water content (*w*_water_) decreases from 13.79% to 1.96%, the total power input (*W*_total_) reduces from 461 kJ to 228 kJ. The power input in the continuous heating period (*W*_heating_) is the main contribution when *w*_water_ ≥7.41%, while the power input in the holding period (*W*_holding_) becomes dominant when *w*_water_ ≤4.76%. The amount of water extracted decreases linearly with the decrease of *w*_water_, and it is strongly related with the power inputs (*W*_heating_ and *W*_holding_). When *w*_water_ decreases from 13.79% to 1.96%, the ratio of power absorbed by water decreases linearly from 38.5% to 7.4%. The energy cost increases from 1.9 W·h/g to 3.9 W·h/g when *w*_water_ decreases from 13.79% to 4.76%. When *w*_water_ is as low as 1.96%, the energy cost increases significantly to 10.0 W·h/g. The water collection ratio is about 30% to 40%. The averaged water collection rate is about 0.84 to 1.57 g/min when *w*_water_ is above 7.41%, and it decreases to about 0.35 g/min when *w*_water_ is below 4.76%.

When a low heating power of 400 W is used, the heating period (*t*_heating_) is prolonged. The power absorption ratios both by water (*Γ*_water_) and by regolith (*Γ*_LRS_) are higher than those at 800 W. Heat is more effectively absorbed by the sample at the low heating power, and less energy is lost to the environment. Then, the energy cost (*η*) reduces to 2.0 to 7.4 W·h/g for the samples with *w*_water_ decreasing from 10.71% to 1.96%. The input power within the total heating time length of 750 s is adequate to dry completely the samples with initial water contents of 4.76% and 1.96%, but it is not adequate for sample 2-3 with a high water content of 10.71%. Residual water content of about 2.0% is shown in sample 2-3. Deceasing the heating power postpones the collection of the first liquid drop to about 300 s. In the following period, the water collection rate and the final water collected ratio are comparable to those obtained at the high power of 800 W.

## Methods

### Lunar regolith simulant

Lunar regolith simulant (LRS) is prepared for massive use purposes. It is known that water ice exists mainly at the polar regions of the Moon. Up to now, the Apollo-16 lunar samples [56,57] were taken from the high-land area of the Moon, which is closest to the polar region. Thus, the present LRS is prepared according to the compositions of lunar regolith at highland areas, and based on the mineral and chemical compositions of Apollo-16 sample (see Table 2). As proved in [56–58], the lunar regolith in the highland area is originally composed of anorthosite, but long-term impact sputtering effect brings other lunar regolith from neighboring mare area. As a result, the lunar regolith in polar regions is composed of both anorthosite and basalt. Two mineral ores on Earth, i.e., the Damiao anorthosite and Jinchuan basalt ores, are selected as raw materials. These ores are crushed mechanically and sieved by different particle sizes. The LRS is prepared by mixing 80% anorthosite and 20% basalt. As shown Table 2, the silicon oxide and the aluminum oxide are major elements. The low Fe composition property is similar to that of the Apollo-16 sample. This is important for the present LSR because Fe component affects the microwave absorption property of the sample.

**Table 2.** Compositions of Apollo sample, raw materials, and the present LRS

Composition	Apollo-16 sample (%)	Anorthosite (Damiao) (%)	Basalt (Jinchuan) (%)	The present LRS (%)
SiO_2_	45.1	65.13	47.79	64.6
Al_2_O_3_	26.8	19.31	16.32	16.8
CaO	15.6	6.42	6.96	3.3
FeO ^a^	5.4	1.29	12.38	3.0
MgO	5.7	0.06	6.89	1.4
TiO_2_	0.60	0.04	1.07	0.5
MnO	0.22	0.01	0.33	0.2
Na_2_O	0.43	4.97	5.39	5.6
K_2_O	0.14	2.76	2.47	3.3
P_2_O_5_	0.10	0.01	0.40	0.1
Total	100.1	100.0	100.0	98.8

^a^ All Fe elements are represented by FeO.

**Fig. 9.**
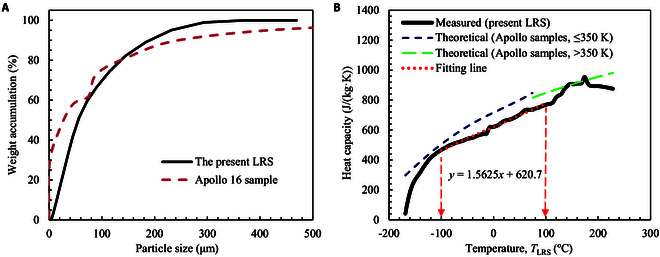
Properties of the LRS. (A) PSD of the samples. (B) Heat capacity of the samples between −170 and 220 °C.

The fine LRS particles below the size of 500 μm are used in this work, and the particle size distribution (PSD) is given in Fig. 9A. The PSD is in the range of 1 to 400 μm with a median grain size (D50) of 55.5 μm. The PSD of Apollo 16 sample [59] is also shown in Fig. 9A. It is an average of 12 subsamples, and the main fractions are 1 to 500 μm. The real density of the present LRS particle is 2.70 g/cm^3^, and the bulk density is 1.47 g/cm^3^ with a calculated porosity of 45.6%.

Heat capacity (*C*_p_) of the LRS is measured through a differential scanning calorimetry (DSC) sapphire method from −170 to 220 °C, and the results are shown in Fig. 9B. Within the temperature range of −100 to 100 °C, the heat capacity of the LRS sample increases almost linearly with the temperature, which is fitted by

$$\begin{array}{c} C_{p}\;\left[J/\left(\mathrm{kg}\cdot K\right)\right]=1.5625\cdot T\;\left[{}^{\circ}C\right]+620.7 \end{array}$$ (7)

where the unit of the temperature is Celsius.

The empirical predictions of heat capacity of the Apollo 14, 15, and 16 samples are given by [60,61], as

$$\begin{array}{c} C_{p}\;\left[J/\left(\mathrm{kg}\cdot K\right)\right]=-23.173+2.1270\cdot T+\left(1.5009\times 10^{-2}\right)\cdot T^{2}\\ -\left(7.3699\times 10^{-5}\right)\cdot T^{3}+\left(9.6552\times 10^{-8}\right)\cdot T^{4},\text{for}\;T\leq 350\;K \end{array}$$ (8)

$$C_{p}\;\left[J/\left(\mathrm{kg}\cdot K\right)\right]=-1,848.5+1,047.41\cdot \text{log}\left(T\right),\text{for}\;T>350\;K$$ (9)

where the unit of temperature is kelvin.

These theoretical relationships are also shown in Fig. 9B. It is seen that the present LSR has a heat capacity very close to that of the Apollo sample.

### Cylinder sample

The sample particles are mixed with water and molded into a cylinder for massive water extraction. The procedures of sample preparation are as follows. The LRS is naturally dry with an intrinsic water below 0.27% ± 0.3%. First, for each sample, the dry particles are weighted to 513 g by an electronic scale. Pure water is added into the dry LSR. The weight of water is determined according to the value of initial water content. As shown in Table 3, 5 different initial water contents from 1.96% to 13.79% are considered, which are below the saturation threshold of water in the compact sample. The weighed pure water and the dry LRS are mixed by the traditional “mud-pie” method with an electric blender (OYD-15). The blender is completely closed during the sample mixing, and little water is lost to the environment. The mixing period is over 30 min to balance water content in the regolith particles. Then, the hydrated LRS with a required weight for each sample is taken from the blender, and it is molded in a stainless steel triaxial mold. The mold has an inner diameter of 70 mm. The density of the sample is controlled by regulating the final height of the sample to a fixed value of 70 mm. Then, a cylinder sample with the size of 70 mm (diameter) × 70 mm (height) is prepared. The calculated bulk density of the cylinder sample is 1.904 g/cm^3^.

**Table 3.** Parameters of dry LRS and icy LSR cylinder samples

Water content of dry LRS	Icy LRS sample cylinders				
	Initial weight, *G*_0_ (g)	Water content, *w*_water_ (%)	Diameter, *D* (mm)	Height, *H* (mm)	Density, *ρ* (g/cm^3^)
0.27% ± 0.3%	513	13.79	70	70	1.904
		10.71			
		7.41			
		4.76			
		1.96			

After molding, the cylinder LRS sample is wrapped by 3 layers of thin plastic sheet to prevent water loss to the environment. The wrapped cylinder is placed into a refrigerator (MELING DW-HL858) where the temperature is fixed at −80 °C. The samples are frozen for over 48 h. By this method, crystalline ice is formed in the LRS [53]. After the sample is frozen, it is taken out again and a small hole is drilled on the top surface of the cylinder sample with a drill bit of 3.3 mm in diameter without taking off the plastic sheet. The depth of the hole is approximately 25 mm. This hole is used to fit the Pt100 temperature sensor, which measures the inner temperature of the sample during the experiment. Then, the sample is placed back into the refrigerator to recover the cryogenic temperature. Since the final weight of the sample is lost by drilling the hole, the sample is reweighted before microwave heating in each experiment.

### Experimental system

**Fig. 10.**
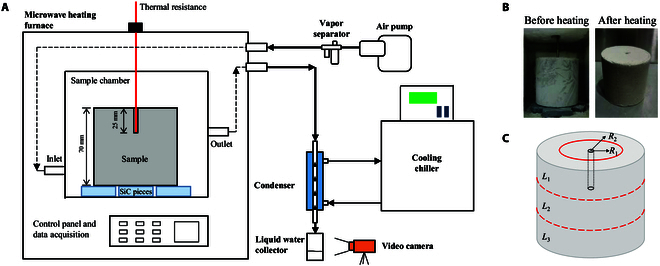
Schematic of the experimental system for microwave heating of icy LRS. (A) Experimental system. (B) Comparison of the sample cylinder before and after microwave heating. (C) Decomposition strategy of the sample cylinder after microwave heating.

An integrated microwave heating unit is built in this study, as shown in Fig. 10A. The microwave heating furnace (ANKS-M12) is able to produce microwaves at 2.45 GHz with a power of 0 to 1,000 W. The LRS sample is placed into the heating chamber, which has a size of 110 mm × 110 mm × 130 mm. A Pt100 temperature sensor (WZPK-291) is set from the top of the chamber, which is then inserted into the small hole of the sample to measure its inner temperature. The temperature range of the sensor is −200 to 500 °C. An inlet tube and an outlet tube are fabricated on the side walls of the chamber, as shown in Fig. 10A. An air pump is used to force air circulation of the chamber through the inlet and outlet tubes. The air flow rate is fixed at 45 l/min. Water vapor in the air and other impurities are separated by a separator at the outlet of the air pump, and clean air is supplied to the sample chamber. With the air pump, the system is operated at positive pressure, and thus, sealing of the system is critical. In the experimental system, the devices are connected by PVC (polyvinyl chloride) transparent hard tubes. The connections are sealed by pneumatic PU (polyurethane tubing) quick connectors, which have good pneumatic seal property. Little gas/vapor escapes from the connection tubes. To reduce leakage of water vapor from the chamber, the chamber walls are sealed by high-temperature resistant sealant, and a ring of rubber pad is used to seal the door of the chamber. It is noted that several pieces of silicon carbide (SiC) are placed into the chamber to protect the microwave generator. The SiC pieces absorb extra microwaves during the experiment, which effectively avoids empty heating once the water content is dried completely. The evaporated water vapor is pumped into a ball condenser with a cooling length of 400 mm. The ball condenser is cooled by a chiller at a temperature of 5 °C. Liquid water is collected after its condensation. The transient water collection process is recorded by a camera (Sony FDR-AX45) at a speed of 25 frames per second. The microwave heating parameters are set in the control panel, including heating power, heating temperature, and holding time. The recorded temperature is monitored by control software and stored in the data acquisition system.

In the experiments, the cooling chiller is set to a cooling temperature of 5 °C. The initial weight of the LSR cylinder, after being taken from the refrigerator, is remeasured by an electronic scale (Yingheng YHC-L01). Microwave heating starts after placing the sample into the chamber and closing the experimental system. It shows that the temperature of the sample increases as heating proceeds until the set value is reached (i.e., the heating period, *t*_heating_). Afterward, the temperature is kept at the set temperature to the end of the experiment (i.e., the holding period, *t*_holding_). The finial weight of the sample is measured to obtain the amount of the water loss by microwave heating (see Fig. 10B). The water collection rate is obtained from the recorded videos by reading the transient volume of collected water with time. Distribution of residual water content in the sample is analyzed by decomposing the cylinder into 6 zones (see Fig. 10C). In the vertical direction, the cylinder is divided into 3 equal levels, i.e., *L*_1_, *L*_2_, and *L*_3_. For each level, the subcylinder is stripped horizontally from outside to inside as 2 rings, i.e., *R*_2_ and *R*_1_. The sample of each zone is crushed. The particles are heated in a thermostatic drying oven for over 2 h at a temperature of 105 °C. The residual water content is obtained by measuring the initial and the final weights of the particles by a high-precision electronic scale (LC-FA1004). Uncertainties of the measured parameters are given in Table 4. The weight of the sample has a maximum uncertainty of 0.1 g, and the monitored temperature has a maximum uncertainty of 1.0 °C.

**Table 4.** Uncertainties of major parameters

Variables	Instruments	Uncertainties
Cylinder mass	Electronic scale (Yingheng YHC-L01)	±0.1 g
Water content	High-precision electronic scale (LC-FA004)	±0.1 mg
Sample temperature	Pt100 temperature sensor	±1.0 °C
Water mass	Electronic scale (Yingheng YHC-L01)	±0.1 g

The experimental cases are given in Table 1. In the first group, 5 samples with the different initial water contents from 1.96% to 13.79% are heated at a high power of 800 W. The heating temperature (*T*_heating_) is set at 100 °C, and the total heating time (*t*_heating_ + *t*_holding_) is set at 750 s. In the second group, the heating power is reduced to 400 W. Three samples with water contents of 1.96%, 4.76%, and 10.71% are studied. With these arrangements, the capability of massive water production of the present microwave heating unit is demonstrated, and at the same time, the 2 most important parameters, i.e., water content and heating power, are included in the present study.

## Data Availability

The data in this study must be obtained through a material transfer agreement (MTA).
